# Opioid-Free Anesthesia for Awake Neurosurgery in a Patient With Asthma and Von Willebrand Disease: A Case Report

**DOI:** 10.7759/cureus.47103

**Published:** 2023-10-16

**Authors:** Heitor Medeiros, Matheus S Nascimento, Luiz Paulo Da Silva Ferreira, Thiago Rocha, Wallace A Da Silva

**Affiliations:** 1 Department of Anesthesiology, Hospital Universitário Onofre Lopes, Natal, BRA; 2 Neurophysiology, Clínica Neurolife, Natal, BRA; 3 Neurological Surgery, Clínica Neurolife, Natal, BRA

**Keywords:** von willebrand disease, glioblastoma, conscious sedation, opioid-free analgesia, awake craniotomy

## Abstract

Anesthesia for awake neurosurgery requires meticulous planning. We report the case of a 44-year-old female with glioblastoma undergoing an awake craniotomy. Due to her asthma and von Willebrand disease, an opioid-free approach was chosen. Conscious sedation was attained using propofol and dexmedetomidine. The operation was successful after nine hours with patient comfort maintained. The patient was discharged from the intensive care unit in two days without sequelae. However, the use of desmopressin caused hyponatremia and cerebral edema. The scalp block was effective for pain management. This case highlights the importance of individualized anesthetic strategies in awake neurosurgeries.

## Introduction

​Anesthesia for neurosurgery represents one of the most challenging branches for an anesthesiologist, given the large variation in the pattern of brain activity and vascular control of the central nervous system among distinct anesthetic drugs [1]. The technique of awake neurosurgery anesthesia involves even more nuances and necessary precautions to ensure comfort, stability, and, above all, safety for the patient and the team involved. An awake craniotomy is a surgical procedure in which the patient is awake for at least a portion of the operation. This approach is used to treat brain lesions that are located near or within areas of the brain that control vital functions, such as speech, movement, and sensation. The goal is to perform the proposed procedure with adequate monitoring in a satisfactory state of consciousness to receive responses during the dynamic intraoperative tests and prevent any motor or sensory deficit during surgery near eloquent areas of the brain.

Moreover, the anesthesiologist’s role in these surgeries is crucial, as they must maintain the patient’s vitals while also allowing for accurate neurological assessment [2]. Factors such as blood pressure, heart rate, and oxygen levels are constantly monitored and adjusted to provide an optimal environment for the brain [3]. The entire process requires a comprehensive understanding of neurophysiology, pharmacology, and specific surgical procedures to effectively navigate these challenges, as the literature does not point to a superior anesthesia technique over another [4].

Along with the surgery’s intricacy, asthma and von Willebrand disease are co-occurring comorbidities that might pose additional risks. Bronchospasms can be triggered in an awake patient by mere anxiety and stress. Furthermore, because of the hemostasis deficit in question, the bleeding might be worse, as well as the cerebral edema caused by its therapy.

The patient discussed in this report provided written informed authorization to publish this case report. This article adheres to the CARE guidelines for reporting case reports [5]. We aimed to report the team’s approach to providing a safe and comfortable experience during awake surgery.

## Case presentation

**Figure 1:**
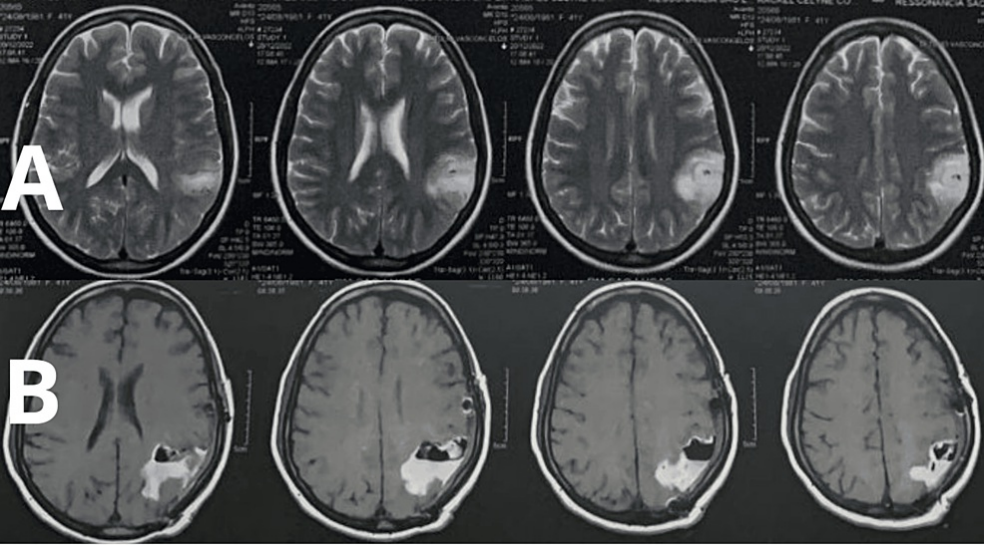
Original image demonstrating magnetic resonance imaging. (A) Before the procedure. (B) One week after the procedure.

A 44-year-old, right-handed, non-smoking, female patient with a medical history of asthmatic bronchitis treated with intermittent doses of salbutamol, hypothyroidism treated with levothyroxine, and von Willebrand disease was admitted to the outpatient neurosurgery department due to seizures refractory to anticonvulsant therapy with phenytoin 200 mg per day. An oligodendroglioma, later confirmed by biopsy, was found in the left parietal region, between the primary motor cortex and Wernicke’s area, measuring 3 x 1.5 x 0.5 cm (Figure 1). Craniotomy and resection guided by neuronavigation were programmed, with a plan to utilize awake anesthesia for continuous monitoring of cognitive capacity and response to neurophysiological stimuli such as language assessment, fluency, association, and calculus.

The patient underwent monitoring, including electrocardiography, peripheral oximetry, and invasive arterial blood pressure measurements. Light sedation was achieved with a target-controlled infusion of propofol, set to an effect-site concentration of 0.7 μg/mL for the execution of a scalp block. The block was performed by administering injections of 4 mL of 0.5% ropivacaine bilaterally to the supraorbital, zygomatic, auriculotemporal, and greater occipital nerves, to a total of 32 mL, utilizing an anatomy-guided technique. We also administered 2% lidocaine without vasoconstrictor, titrated, directly into the dura after opening to a total of 6 mL.

Conscious sedation was accomplished via a continuous infusion of dexmedetomidine at a dose of 0.5 µg/kg/hour and propofol set to an effect-site concentration of 0.8 μg/mL. During the procedure, a neurophysiologist conducted a dynamic assessment of the following parameters: comprehension, naming, calculation, reading, writing, stereognosis, graphesthesia, tactile extinction, and visual extinction. Aminocaproic acid at a dose of 50 mg/kg every four hours and desmopressin at a dose of 0.3 µg/kg every eight hours were started a day before and maintained during the surgery, as recommended by the assistant hematologist for the case. Sodium levels were 142 mg/dL the day prior, and subsequent hyponatremia of 128 mg/dL was noted in the first serum measurement in the intraoperative period. Clinically significant cerebral edema was noted after opening the dura mater, which was treated with 400 mL of 3% hypertonic saline in four hours and mannitol 0.5 g/kg. Improvement of the edema occurred in the last two hours of the surgery after a measured sodium level of 136 mg/dL.

Throughout the duration of the procedure, we used the monitored anesthesia care technique, and the patient reported neither pain nor discomfort. The patient remained in a state of conscious sedation, lightly sleeping but quickly responsive when prompted, and capable of maintaining interaction with the professional administering intraoperative cognitive tests. The patient’s responses were consistent with satisfactory implementation of the anesthetic plan and comfort.

Except for the refractory hyponatremia and cerebral edema, the procedure was completed without any other complications. Upon completion of intracranial resection of the tumor, roughly four hours into the procedure, the target propofol was adjusted to an effect-site concentration of 1.2 μg/mL. This adjustment induced deeper sedation, allowing the patient to sleep through the remainder of the surgery and awaken only during the final stages of the procedure, approximately two hours later. Upon admission to the intensive care unit, the patient showed no neurological alterations for the next two days. However, later, the patient exhibited partial aphasia for seven days before returning to normal and repeating the MRI (Figure 1), which was expected considering the procedure and the cerebral edema afterward. She received dexamethasone 4 mg every six hours after the surgery and was discharged with oral prednisolone for the next month.

## Discussion

Matching patient needs with surgical choices can lead to better options and fewer complications. The decision to prefer regional anesthesia techniques and not to use opiates was consistent with the idea of avoiding causing respiratory depression in an asthmatic individual who needed to stay awake. On the other hand, the requirement to optimize coagulation with desmopressin due to von Willebrand disease caused cerebral edema as variations in sodium plasma concentration are part of the medication mechanism, resulting in substantial discomfort for the present surgery.

Awake craniotomy has emerged as the main technique for surgically resectioning gliomas in eloquent areas, with the main advantage of enabling real-time monitoring of cortical and subcortical functions without necessarily increasing the patient’s stress or anxiety, resulting in better neurological and perioperative outcomes [6]. While sustaining the same surgical implications as any neurosurgery, it brings the additional challenge of the need for light sedation and pain management with the maintenance of consciousness. The main anesthetic techniques available for this kind of surgery include monitored anesthesia care, asleep-awake-asleep, and the two-step anesthesia technique (asleep-awake) [7]. The second was chosen because it does not require airway management, as did the first option, which was convenient due to the increased risk of airway bleeding and asthma-related bronchospasms, which could also worsen cerebral perfusion by diminishing venous return.

An important analgesia strategy, used with success in this case, was the scalp block. As pinning causes an important painful stimulus, even under general anesthesia, this regional anesthesia technique provides better hemodynamic control during the procedure, as well as postoperatively proving superior to the local anesthetic infiltration technique [8]. Furthermore, it was chosen for its ease and safety of execution as well as its opioid-sparing properties, complementing the opioid-free and multimodal strategies [9].

The opioid-free route has some major advantages. For asthmatics, a lower incidence of bronchospasm and respiratory depression is associated with better postoperative pulmonary function [10]. As a non-smoking woman, it likely reduced postoperative nausea and vomiting (PONV) [11]. Furthermore, without opioid synergism with other sedatives, maintaining the patient awake is safer and easier, with improved patient compliance and communication during cortical mapping [12].

Total intravenous anesthesia is becoming more popular in neurosurgery for various reasons [13]. The main advantages are related to propofol, which, in contrast to inhalation drugs, can reduce both cerebral blood flow and intracranial pressure while maintaining flow-metabolism coupling [14]. It is also an excellent choice for lowering the risk of PONV and for neuromonitoring while allowing for a smooth induction and fast emergence if necessary [15].

Dexmedetomidine, on the other hand, is a sympatholytic that works as an agonist of alpha-2-adrenergic receptors and is often employed as a neuroprotective adjuvant to anesthetics in neurosurgery [16]. Furthermore, there is no possibility of respiratory depression, which reduces the need for further sedatives that would increase it, which was undesirable in this circumstance [16].

Our patient also had type 1 von Willebrand disease, the most prevalent inherited bleeding disease that requires individualized perioperative care [17]. Desmopressin is the first-line treatment for this condition and should be administered before any surgical treatment. Antifibrinolytic medicines, such as aminocaproic acid, may also bind to fibrinogen’s lysine sites and improve clot stabilization, lowering the risk of bleeding [18].

Desmopressin is a synthetic vasopressin receptor agonist that causes renal water retention, resulting in hyponatremia, which in this case led to clinically significant cerebral edema. Despite this, the therapy may not be stopped during the surgery, and intravenous hypertonic saline solution should be administered [19].

## Conclusions

Future large-scale studies should focus on awake craniotomy anesthesia as well as its relationship with the most prevalent comorbidities that could hinder its success, as asthma and von Willebrand disease did in this case. This could bring unique opportunities to advance patient care in neurosurgery, with critical insights into the specific anesthetic needs and challenges of different patients, potentially leading to the development of tailored anesthesia protocols. They could also contribute to our understanding of how these conditions interact with awake craniotomy and its several techniques. However, the success of these studies will require rigorous study design, careful patient selection, and close collaboration between neurosurgeons, anesthesiologists, and other specialties, such as hematologists or pneumologists in this case. As we anticipate these advancements, it is clear that the future of awake craniotomy anesthesia research holds promising prospects for improving patient outcomes in this complex surgical procedure.
